# Study of effect of guided meditation on quality of life in patients of end stage renal disease (ESRD) on maintenance hemodialysis – a randomised controlled trial

**DOI:** 10.1186/s12906-022-03717-8

**Published:** 2022-09-09

**Authors:** Bhalendu S. Vaishnav, Jekishan Jayeshbhai Hirapara, Maulin K. Shah

**Affiliations:** 1 Department of Medicine, H M Patel Centre for Medical Care and Education, Pramukh Swami Medical College, Shree Krishna Hospital, Bhaikaka University, Karamsad, Gujarat India; 2 Present Address- Privilege Centre, The Healing tree, Shree Krishna Hospital, Gujarat 388325 Karamsad, India

**Keywords:** Chronic kidney disease, Maintenance hemodialysis, Guided meditation, Quality of life

## Abstract

**Background:**

There is paucity of data regarding effects of guided meditation (Yoganidra) on quality of life among patients of chronic kidney disease on maintenance hemodialysis. Our objective was to study effects of guided meditation on physical, emotional, and cognitive dimensions of well-being and quality of life in patients undergoing maintenance hemodialysis.

**Method:**

We collected baseline and post intervention data in control and intervention groups on hemodialysis and studied the effect of Guided mediation provided for 6 weeks.

**Result:**

Eighty patients (forty in control and intervention group each) were studied. Mean age was 51 years. Hypertension and Diabetes were the most common etiological condition (28.75%) followed by undermined aetiology (25.00%). 8.75% of the patients had dialysis vintage of less than 1 year. There was statistically significant difference in qualities of happiness and all measures of physical general wellbeing. There was statistically significant difference in burden and effect of kidney disease as well as symptoms of kidney disease post intervention in Kidney Disease Quality of Life score. We carried out Qualitative analysis in our study by maintaining a diary of their subjective experiences related to listening music/guided meditation during the study period in which the feeling of peace and feeling inspired to manage the illness/ do regular work were the most common experiences (97.5%) reported by participants.

**Conclusion:**

Guided meditation resulted in statistically significant improvement in happiness, enthusiasm, inspiration, activeness, alertness, awareness, degree of stability, self-confidence, clarity of thoughts, control over anger, self-reflection intervention in the intervention group. It reduced perceived stress. It improved burden and effect of kidney disease, symptoms of kidney disease and total Kidney Disease Quality of Life score. In qualitative dimensions of wellbeing (as emerging from analysis of results of diary), feeling of peace and feeling inspired to manage the illness/ continue regular work, clarity of thoughts, happiness, concentration, reduction of laziness, improved sleep pattern, reduction in anger among other psychological components.

**Trial registration:**

This trial has been registered under clinical trial registry of India. (CTRI number-CTRI/2020/02/023438) (Date: 19/02/2020).

**Supplementary Information:**

The online version contains supplementary material available at 10.1186/s12906-022-03717-8.

## Background

The pattern of disease burden in the twenty-first century has significantly shifted towards chronic diseases (CDs) [1]. Population aging and lifestyle-modifiable risk factors, accompanied by a decline in early-life infectious diseases, have resulted in the emergence of CDs as a major global health threat [2]. Among CDs, Chronic kidney disease (CKD) is of particular significance and contributes heavily to the global CVD and end-stage renal disease (ESRD) [3, 4]. It is estimated that 1, 00,000 new patients of end stage renal disease (ESRD) enter renal replacement programs annually in India [5]. CKD patients are often confronted with limitations in food and fluid intake; with physical symptoms such as itching and lack of energy; with psychological stressors such as loss of self-concept and self-esteem, feelings of uncertainty about the future, and feelings of guilt towards family members; and with problems in the social domain [6–8].

Health-related quality of life (HRQOL) is a critically important outcome for patients with end-stage renal disease (ESRD) [9]. The National Quality Forum selected the Kidney Disease Quality of Life Short-Form survey (KDQOL™-36) as the tool of choice for assessing this outcome in adult patients with ESRD [10]. Complementary practices or alternative medicines are now widely known and practiced as adjuvant therapy in global health care systems to enhance overall health and well-being of the patients [11]. Meditation has been also shown to decrease anxiety and stress by increasing the tolerance level to pain and improve wellbeing and QOL of patients with chronic diseases [12]. In the present era, meditation has been considered as a potential intervention to decrease stress and anxiety and is gaining popularity over the past few decades. Meditation is a form of a mind–body intervention as it helps to influence the mind to adapt to the body’s clinical symptoms through mechanisms of the parasympathetic nervous system and the decrease in stress hormone levels [13]. In the parlance of ancient Indian psychology, well‑being refers to awareness and manifestation of a transcendental state of awareness, which human beings are capable of accessing [14]. Yoga is the science, the process, the effort, and action by which man attempts to pass out of the limits of his ordinary mental consciousness into a greater spiritual consciousness, from the phenomenal to the real man [15]. Yoganidra, a part of Pratyahara technique of Yoga, facilitates a state of inner awareness and outer detachment; leading oneself to relax completely and consciously which can even potentiate many self‑development and well‑being practices. Yoganidra is one of the earliest Indian techniques which allows one to develop an experiential state of simultaneous relaxation and detachment [16]. We therefore set out to study effects of Yoganidra on various dimensions of well-being such as perceived stress, happiness, quality of life and psychological general well-being along with biochemical parameters amongst patients of CKD on MHD.

## Objectives

- 1. To study physical, psychological, and biochemical parameters of patients of CKD undergoing maintenance dialysis.
- 2. To study Health related quality of life in CKD patients. (KDQOL).
- 3. To document effects of dynamic meditation (Yoganidra) on KDQOL and psychological wellbeing through quantitative and qualitative observations.

## Methods

### Study design

After an informed written consent as attached in Annexure for 80 patients undergoing hemodialysis were enrolled in the study (40 in intervention group, 40 in control group) using balanced double-blinded (participants and care provider) randomisation method as suggested by statistician.( This trial registered under Clinical Trial Registry of India(CTRI): CTRI/2020/02/023438).

### Study setting

Patients of CKD on hemodialysis attending Shree Krishna Hospital, Karamsad.

#### Inclusion criteria

- 1. Age between 18–70 years.
- 2. CKD with eGFR (by CKD-EPI equation) < 15 ML/MIN/1.73 m^2^ (KDIGO definition) and on maintenance hemodialysis 3 times a week.
- 3. Patients who are clinically stable (not having suffered from any acute illness) for last 3 months.

#### Exclusion criteria

- 1. Any solid organ or bone marrow transplant recipient.
- 2. Active malignancy in last 2 years.
- 3. Palpitation or dyspnoea at rest or asymptomatic only when resting.
- 4. Ethnicity: Non-Indian.
- 5. Pregnancy (in case of female).
- 6. Current immunosuppressive drug therapy.
- 7. Expected life expectancy < 1 year [17]

### Methodology

Consent, Randomization: An informed consent was obtained from all participants.

- Group 1: Intervention group.
- Group 2: Control group.
- 1.Pre and post intervention Evaluation:
  - Clinical and Laboratory Evaluation: Symptoms, General and systemic examination, Type and detail Of Hemodialysis received, Assessment of Quality/ Effectiveness Of hemodialysis, Baseline and End of study biochemical parameters (Histogram, Creatinine, Urea, Electrolytes, Calcium, Phosphorus)
  - KDQOL, (Gujarati-vernacular /local language)
  - Experimental Record (Diary)
  - Perceived stress scale [18]
  - Faces scale of Happiness (Andrews) [19]
  - VAS Scale for Stress [20]
  - Investigator designed scale for assessment of positive wellbeing

## Intervention

- Intervention group: Guided Meditation session of 30 min during each dialysis (three days /week)
- Control group: Standard quality care
- Clinical and biochemical record of patients were maintained during the study period as per standard practices as advised by Nephrologist (clinical assessment at each dialysis visit and laboratory investigations every month as per standard protocol).
- All forty participants in the intervention group were asked to write whatever they experienced so far as effects of Yoganidra on physical and emotional wellbeing were concerned.
- Duration of intervention: 6 weeks (21/02/2020 to 03/04/2020)
- An audio recording of guided dynamic meditation session: was prepared by the principle /co-investigator having experience of conducting similar sessions in diverse populations.

## Ethical approval

This study was approved by the Institutional ethical committee-2, H M Patel centre for medical care and education, Karamsad, Anand (IEC/HMPCMCE/114/Faculty/246/19) approved on 18/12/2019 and also registered under clinical trial registry of India. CTRI number-CTRI/2020/02/023438. (Date:19/02/2020).

### Statistical analysis

• The quantitative variables are expressed as Mean ± SD. Qualitative variables are expressed as frequencies/ percentages and compared between groups using Fisher Exact test.

*P*-value < 0.05 was considered statistically significant. STATA version 14.2 and Microsoft Excel software were used for tabulation of data and statistical analysis. Pre and post intervention comparison of clinical, QOL parameters, qualitative analysis (thematic analysis of subjective experiences).

### Primary outcome variable

- 1. Change in Quality of Life as measured by KDQOL.
- 2. Psychological general wellbeing as measured by quantitative and qualitative methods

## Results

### Baseline and post-intervention clinical and biochemical characteristics

**Fig. 1:**
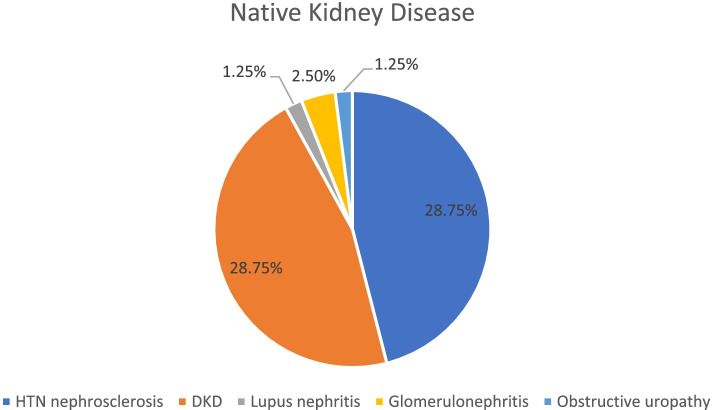
Etiology of native kidney disease

The mean age of study population was 51 years with SD of 16.49 as shown in Table 1. The gender distribution (M/F) was 53(66.25%)/27(33.75%). 32.50% of patient were farmers and housewife. Hypertension and Diabetes (HTN and DM) were the most common etiological condition (28.75%) followed by CKD of undermined aetiology (25.00%) as shown in Fig. 1. HTN, DM and ischemic heart disease (IHD) were present in 98.25/78.75/77.50% respectively. 18.75% of the patients had dialysis vintage of less than 1 year. The Demographic Data and Laboratory parameters in control and intervention groups were comparable. There was statistically significant improvement in S. Potassium, S. Phosphate, S. Protein, S. Uric acid in the intervention group. There was no statistically significant change in KT/V ratio in the intervention groups at the beginning and at the end of study period as shown in Table 2.

**Table 1:** Demographic data of intervention and control group

Parameter		Group A (IG)	Group B (CG)
Age	15–30 years	9(22.50)	4(10.00)
	31–45 years	6(15.00)	8(20.00)
	46–60 years	9(22.50)	17(42.50)
	61–75 years	15(37.50)	11(27.50)
	76–90 years	1(2.50)	-
Gender	Male	24(60.00)	29(72.50)
	Female	16(40.00)	11(27.50)
Socio economic status	Rural	27(67.50)	28(70.00)
	Urban	13(32.50)	12(30.00)
Occupation	Unemployed	3(7.50)	2(5.00)
	Student	6(15.00)	3(7.50)
	Businessmen	3(7.50)	3(7.50)
	Farmer	10(25.00)	16(40.00)
	Housewife	13(32.50)	13(32.50)
	Retired	3(7.50)	2(5.00)
	Teacher	1(2.50)	1(2.50)
	Driver	1(2.50)	-
Native kidney disease	Undetermined	11(27.50)	9(22.50)
	HTN Nephrosclerosis	17(42.50)	16(40.00)
	DKD	9(22.50)	14(35.00)
	Lupus nephritis	1(2.50)	-
	Glomerulo- nephritis	1(2.50)	1(2.50)
	Obstructive uropathy	1(2.50)	-
Hypertension	Yes	39(97.50)	38(95.00)
	No	1(2.50)	2(5.00)
DM-II	Yes	31(77.50)	32(80.00)
	No	9(22.50)	8(20.00)
IHD	Yes	30(75.00)	32(80.00)
	No	10(25.00)	8(20.00)
Dialysis vintage	< 1 year	9(22.5)	6(15)
	> 1 year	31(77.5)	34(85)

**Table 2:** Comparison of Laboratory parameters in Intervention and Control group

Sr. No	Parameter		Intervention		Control(M ± SD)	
			(M ± SD)	*P*-value	(M ± SD)	*P*-value
1	Hb	Pre	9.85(1.76)	0.2232	10.90(1.43)	0.1617
		Post	10.19(1.54)		10.59(1.40)	
2	TC	Pre	6.65(1.91)	0.2498	7.10(1.95)	0.8140
		Post	6.24(1.94)		7.03(1.91)	
3	Platelets	Pre	212.65(59.03)	0.2612	216.97(64.87)	0.7965
		Post	203.1(51.11)		214.75(74.62)	
4	Urea	Pre	79.67(25.88)	0.2958	97.37(33.26)	0.0546
		Post	84.02(23.65)		88.15(28.08)	
5	Creatinine	Pre	7.77(2.49)	0.6804	9.00(2.93)	0.1717
		Post	7.90(2.41)		8.52(2.45)	
6	S.Na	Pre	137.32(3.93)	0.8291	136.37(3.62)	0.6926
		Post	137.17(3.21)		136.65(3.46)	
7	S.K	Pre	4.84(0.68)	0.0408	5.35(0.87)	0.2176
		Post	5.18(0.85)		5.48(0.94)	
8	S.Cl	Pre	103.27(3.79)	0.9041	101(8.89)	0.5957
		Post	103.2(3.48)		101.85(3.56)	
9	S.HCO3	Pre	24.95(3.51)	0.5102	23.56(4.15)	0.1621
		Post	24.47(4.89)		24.59(3.40)	
10	S. Ca	Pre	7.80(1.21)	0.7581	8.16(0.79)	0.4040
		Post	7.74(0.63)		8.26(0.70)	
11	S.PO4	Pre	3.66(1.27)	0.0113	4.62(0.92)	0.0916
		Post	4.16(1.42)		4.97(1.50)	
12	S. Protein	Pre	6.96(0.70)	0.0170	7.17(0.73)	0.0097
		Post	6.70(0.48)		6.80(0.63)	
13	S.Albumin	Pre	3.4(0.88)	0.2367	3.50(0.50)	0.0014
		Post	3.20(0.64)		3.26(0.51)	
14	Uric acid	Pre	5.25(1.36)	0.0194	5.84(1.80)	0.6236
		Post	5.96(1.78)		5.95(1.38)	
15	S.Iron	Pre	72.8(35.84)	0.3371	73.7(35.37)	0.9648
		Post	65.85(36.27)		73.95(36.28)	
16	TIBC	Pre	198.275(46.07)	0.3802	210.8(43.67)	0.9300
		Post	192.8(50.57)		210.275(42.18)	
17	S.ferritin	Pre	896.68(547.90)	0.4940	833.64(396.21)	0.3857
		Post	849.85(421.56)		786.97(392.90)	
18	Single pool KT/V	Pre	1.76(0.34)	0.3320	1.86(0.39)	> 0.995
		Post	1.78(0.30)		1.86(0.39)

### Baseline and post intervention results of psychological wellbeing

**Fig. 2:**
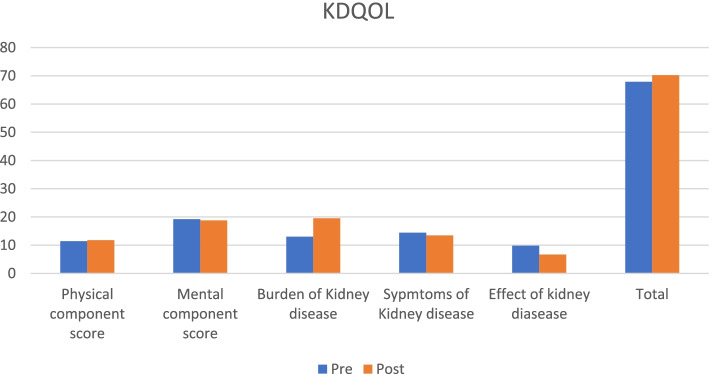
Components of KDQOL

Baseline degree of psychological measures studied were comparable in control and intervention groups. There was significant difference in the degree of happiness measured by Faces scale and perceived stress post-intervention (*p* = 0.0027 and *p* =  < 0.001 respectively). There was no significant reduction in degree of stress measured by VAS scale. There was statistically significant improvement in the qualities of enthusiasm, happiness, feeling inspired, activeness, alertness, awareness, stability, self-confidence, clarity of thoughts, control over anger, soul inspection except Quietness in intervention group in pre and post study as shown in Table 3. There was significant improvement in Burden of Kidney disease (*p* =  < 0.001) and Effect of Kidney disease (*p* = 0.0001) as well as Symptoms of kidney disease (*p* = 0.01461) in the KDQOL score post intervention. There was significant improvement in total KDQOL score in pre and post study in intervention group as shown in Table 4 and Fig. 2.

**Table 3:** Comparison of qualitative parameters between intervention and control group

Parameter		Intervention		Control	
		(M ± SD)	*P*-value	(M ± SD)	*P*-value
Faces scale for Happiness	Pre	1.87(0.93)	0.0027	1.77(0.92)	0.0831
	Post	1.32(0.52)		1.69(0.85)	
VAS Scale for stress	Pre	1.5(1.10)	0.3310	1.5(0.94)	0.7111
	Post	1.3(0.51)		1.47(0.65)	
Positive well-being					
Enthusiastic	Pre	3.8(0.85)	< 0.001	3.77(0.63)	0.4219
	Post	4.75(0.43)		3.83(0.69)	
Quiet	Pre	3.95(0.63)	> 0.995	3.80(0.40)	> 0.995
	Post	4.92(0.26)		3.80(0.40)	
Happy	Pre	3.92(0.69)	< 0.001	3.97(0.65)	0.1032
	Post	4.9(0.30)		4.08(0.69)	
Inspired	Pre	3.75(0.77)	< 0.001	3.88(0.70)	0.3242
	Post	4.77(0.42)		3.86(0.72)	
Active	Pre	3.77(0.89)	< 0.001	4.05(0.79)	> 0.995
	Post	4.67(0.47)		4.05(0.79)	
Alert	Pre	3.75(0.92)	< 0.001	3.91(0.69)	> 0.995
	Post	4.65(0.48)		3.91(0.69)	
Awareness	Pre	3.82(0.90)	0.0001	3.94(0.62)	> 0.995
	Post	4.65(0.48)		3.94(0.62)	
Stability	Pre	3.77(0.80)	0.0001	3.94(0.53)	> 0.995
	Post	4.47(0.50)		3.94(0.53)	
Self confidence	Pre	3.87(0.85)	< 0.001	3.88(0.62)	> 0.995
	Post	4.62(0.49)		3.88(0.62)	
Clarity of thought	Pre	3.85(0.80)	< 0.001	3.91(0.64)	> 0.995
	Post	4.75(0.43)		3.91(0.64)	
Control over anger	Pre	3.67(0.72)	< 0.001	3.75(0.60)	> 0.995
	Post	4.92(0.26)		3.75(0.60)	
Soul inspection	Pre	3.9(0.74)	< 0.001	3.86(0.59)	> 0.995
	Post	4.75(0.70)		3.86(0.59)	
Perceived Stress Scale	Pre	10.82(4.15)	< 0.001	11.22(4.00)	> 0.995
	Post	2.95(5.15)		11.22(4.00)

**Table 4:** KDQOL

Parameter		Intervention		Control	
		(M ± SD)	*P*-value	(M ± SD)	*P*-value
KDQOL					
i) Physical component score (PCS)	Pre	11.45(1.29)	0.1809	11.38(0.99)	0.6611
	Post	11.77(0.73)		11.47(0.84)	
ii)Mental component score (MCS)	Pre	19.22(1.77)	0.1509	19.25(1.69)	0.3611
	Post	18.75(1.08)		19.05(1.73)	
iii)Burden of Kidney Disease	Pre	13(6.81)	< 0.001	15.02(6.66)	0.0536
	Post	19.52(1.89)		15.91(4.93)	
iv)Symptoms of kidney disease	Pre	14.42(2.22)	0.01461	14.88(3.20)	0.1069
	Post	13.45(0.74)		15.33(2.84)	
v)Effect of kidney disease	Pre	9.8(4.32)	0.0001	9.61(3.68)	0.0736
	Post	6.7(0.82)		9.97(3.73)	
Total	Pre	67.9(6.20)	0.0355	69.82(6.49)	0.1265
	Post	70.2(1.95)		64.57(22.36)

### Qualitative analysis of results of diary

**Fig. 3:**
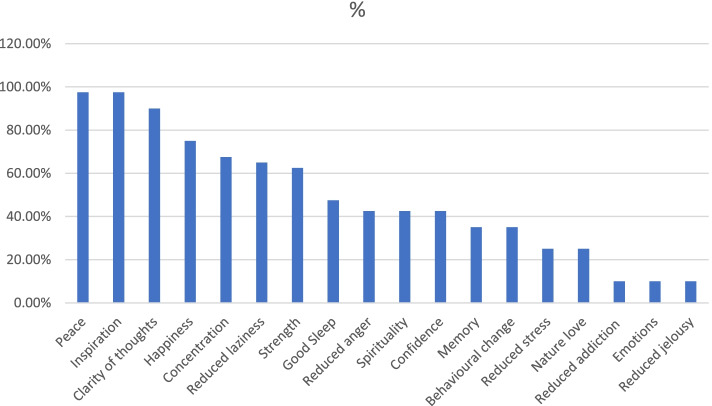
Qualitative analysis of diary

All forty participants in the intervention group submitted their diaries for qualitative analysis**.** Most of the responses were in form of short sentences, sometimes simple words and were explicitly thematic, as it were, such as freshness, happiness, peace, etc. [21] By and large none wrote descriptive notes. Each diary contained average 10 reflective observations. There were no illegible records. After familiarizing with the data, two codes/categories were generated to classify responses, viz. physical well-being and psychological well-being. The responses pertaining to physical wellbeing included reduction in fatigue, feeing more energy, improvement in sleep. Responses pertaining to psychological wellbeing consisted of happiness, reduction in laziness, stress and anger, improvement in memory, confidence, etc. It was observed that saturation of reflections was reached during third week. Observations were analysed independently by two investigators, whose analysis was similar. There was concurrence between some of the qualitative and quantitative dimensions of wellbeing. The frequency of patients (%) who expressed various feelings/experiences is shown in the Fig. 3.

## Discussion

As a first step in our study, we analysed clinical, biochemical and HRQOL characteristics of patients of MHD in CKD. We found that they are by and large in conformity with national studies carried out on this aspect in much larger cohorts. Yoganidra intervention significantly reduced stress, increase in happiness, and overall QOL in our study. Assessment of stress using VAS is an accepted and reliable method which avoids communication‑related issues encountered in impersonal questionnaire‑based studies [22]. The Faces scale for happiness has been shown to be reliable in predictive value and reflects totality of one’s state of happiness. Reduction in stress and anxiety levels and definite increase in general well-being of adolescents as well as in trainee teachers as a result of Yoganidra intervention have been observed by Vaishnav BS et al. [23, 24]. Parker et al. demonstrated that there are conceptual difference and practical distinction between Yoganidra and other methods of guided relaxation [25]. Elaboration of deeper and esoteric significance of effects of Yoganidra provided in standard ancient scriptures is beyond the scope of this study. The neural circuits that underlie each of these constituents overlap partially and thus can be transformed through experience and training [26].

Rajendra Kumar Pandey et al. in his study of Effects of 6 months yoga program on renal functions and quality of life in patients suffering from CKD in 54 patients have observed that there was significant reduction in systolic and diastolic blood pressure, blood urea and serum creatinine levels, and significant improvement in physical and psychological domain of the World Health Organization QOL (as assessed by BREF QOL scores) were seen after 6 months [27].

In a study of 175 patients, Sapna Kothari et al. have reported that meditation results in significant decrease in depression, anxiety and stress in hemodialysis patients practicing Arham Purushakar meditation, and also improvement in other physical and medical conditions [28].

Masina et al., (2016) have reported that overall health related quality of life was low (mean score 59.9 ± 8.8, maximum possible score 100) with the lowest scores recorded for physical health component summary score (50.4 ± 22.8) compared to mental health component summary (61.3 ± 23.0) and kidney disease component summary (67.9 ± 13.2) and also reported that low household income (< $4000 per year) was associated with lower mental health component scores [29].

We have studied happiness, stress, positive wellbeing parameters, psychological wellbeing, and KDQOL-36 questionnaire. The Uniqueness of our study is that we have done qualitative analysis- Experiential effects of Guided meditation” (a researcher designed tool for capturing certain positive and deeper qualities of one’s well‑being which are not hitherto overtly covered). Attributes measured were feelings of enthusiasm, being active, alert, and inspired, experiencing quietude, experiencing a state of self‑awareness, stability and self‑confidence, having clarity of thoughts and control over anger.

Thus, in a small set of study participants all parameters of general wellbeing in CKD patients were taken into consideration and results were analysed accordingly giving us an insight on effect Yoganidra. Summing up the effect of Yoganidra on patients having CKD with MHD, it had positive impact on various factors studied in our study like happiness, stress, positive wellbeing, laboratory parameters as well in experimental effects.

### Limitations

Longer duration of intervention (> 6 weeks), larger sample size and long-term follow-up, and external validation of Investigator designed tool can enhance strength of the intervention. Implementation of Yoganidra for longer period may result in better control of blood pressure. The scientific pursuit can be further enhanced by studying effects of guided meditation on physiological and biochemical parameters such as heart rate variability and markers of inflammation.

## Conclusion

Guided meditation resulted in statistically significant improvement in qualitative dimensions of wellbeing such as degree of happiness, enthusiasm, inspiration, activeness, alertness, awareness, degree of stability, self-confidence, clarity of thoughts, control over anger, self-reflection in the intervention group as measured by quantitative and qualitative methods. There was significant improvement in stress level as measured by perceived stress scale, KDQOL related to burden and effect of kidney disease, symptoms of kidney disease and total KDQOL score. Guided meditation is easy to implement, and effective in enhancing several components of quality of life and wellbeing; it has high acceptability and compliance. It can be easily incorporated in standard of care of patients of CKD on MHD. It can be recommended for inclusion in standard of care for enhancement of quality of life of patients on maintenance hemodialysis.

## Supplementary Information

**Additional file 1.** 

## Data Availability

All data generated or analysed during this study are included in this published article [and its supplementary information files].

## Abbreviations

CD: Chronic Diseases
CKD: Chronic Kidney Disease
CVD: Cardiovascular disease
DM: Diabetes Mellitus
ESRD: End Stage Renal Disease
eGFR: Estimated Glomerular Filtration Rate
HRQOL: Health Related Quality Of Life
HTN: Hypertension
IHD: Ischemic Heart Disease
KDIGO: Kidney Disease Improving global outcome
KDQOL: Kidney Disease Quality of Life
MCS: Mental Composite Score
MHD: Maintenance Hemodialysis
PCS: Physical Composite Score
SD: Standard Deviation
QoL: Quality of Life
VAS: Visual Analogue Scale
